# Anesthesia Management for Pediatrics with Congenital Heart Diseases Who Undergo Cardiac Catheterization in China

**DOI:** 10.1155/2021/8861461

**Published:** 2021-01-30

**Authors:** Chun-Mei Xie, Yun-Tai Yao

**Affiliations:** ^1^Department of Anesthesiology, Fuwai Yunnan Cardiovascular Hospital, Affiliated Cardiovascular Hospital of Kunming Medical University, Kunming 650000, China; ^2^Department of Anesthesiology, Fuwai Hospital, National Center for Cardiovascular Diseases, Peking Union Medical College and Chinese Academy of Medical Sciences, Beijing 10037, China

## Abstract

**Objectives:**

The goal of this study was to summarize anesthesia management for pediatrics with congenital heart diseases who undergo cardiac catheterization procedure in China.

**Methods:**

The relevant articles were identified through computerized searches in the CNKI, Wanfang, VIP, and PubMed databases through May 2020, using different combinations of keywords: “congenital heart diseases,” “pediatric,” “children,” “anesthesia,” “cardiac catheterization,” “interventional therapy,” “interventional treatment,” “interventional examination,” and “computed tomography.”

**Results:**

The database searches identified 48 potentially qualified articles, of which 25 (9,738 patients in total) were determined to be eligible and included. The authors collect data from the article information. Anesthesia methods included endotracheal intubation or laryngeal mask ventilation general anesthesia, monitored anesthesia care, and combined with sacral canal block. Anesthesia-related complications occurred in 7.41% of the patients and included dysphoria, respiratory depression, nausea, vomiting, cough, increased respiratory secretion, and airway obstruction. The incidence of procedure-related complications was 12.14%, of which the most common were arrhythmia and hypotension.

**Conclusions:**

For pediatric patients with congenital heart diseases who undergo cardiac catheterization procedures in China, arrhythmia and hypotension are the most common procedure-related complications. Monitored anesthesia care is the commonly used anesthesia methods, and dysphoria, cough, nausea, vomiting, and respiratory depression are frequent complications associated with anesthesia.

## 1. Introduction

Congenital heart diseases (CHDs) are the most common form of congenital abnormality. Marelli et al. [1] reported that CHD occurs in over 1% of newborns, and the morbidity is similar in China. Cardiac catheterization is an integral part of medical management for pediatric patients with CHD [2]. Cardiac catheterization and angiography were established and widely used by the 1950s [3]. Cardiac catheterization can avoid the trauma of thoracotomy and the potential risk of cardiopulmonary bypass (CPB), eliminate the possible adverse consequences of CPB, and significantly shorten the length of hospital stay. Cardiac catheterization for CHD has developed rapidly in recent years, and the main emphasis has shifted toward innovative therapeutic interventions [4]. These advances allow children with CHD many additional nonsurgical options, increase patients' surgery-free intervals, and can possibly postpone or even replace open heart surgery. Thus, cardiac catheterization has gradually become the preferred and most common method for treating children with CHD. However, the optimal anesthesia method for cardiac catheterization of CHD in children is still controversial. This paper included articles published in various regions of China on cardiac catheterization of CHD in children, in order to find out the optimal anesthesia method and summarize the anesthesia experience and complications.

## 2. Methods

### 2.1. Search Strategy

Relevant articles were identified through computerized searches in the CNKI, Wanfang, VIP, and PubMed databases through May 2020 using different keywords combinations including “congenital heart diseases,” “pediatric,” “anesthesia,” “children,” “cardiac catheterization,” “interventional therapy,” “interventional treatment,” “interventional examination,” and “computed tomography.” The inclusion criteria mainly focused on retrospective articles and controlled studies. The exclusion criteria included the following: ① studies published before 2000, ② duplicate publication, ③ a sample size of fewer than 100 cases, ④ conference abstracts, and ⑤ studies that lacked details.

Both authors (C. M. X and Y. T. Y) independently reviewed the titles and abstracts of all the candidate reports for eligibility and excluded those that were obviously ineligible. The eligibility of the remaining reports for final inclusion was subsequently determined by examining the full text.

### 2.2. Data Abstraction

The following data from the included articles were abstracted to a data collection form by each author independently: ① author, year, journal of publication, and research institutions; ② total number of patients, sex, age, weight, operation methods, anesthesia methods, method of airway administration, and drugs; ③ intraoperative monitor; ④ anesthesia- and procedure-related complications. Disagreements were resolved by discussion between both authors during the data abstraction process.

## 3. Results

### 3.1. The Articles

**Figure 1:**
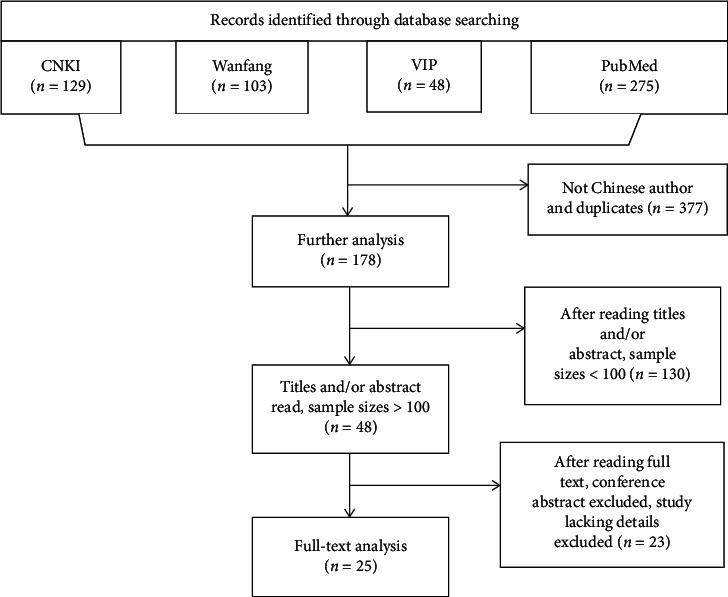
Flow diagram of study selection.

**Figure 2:**
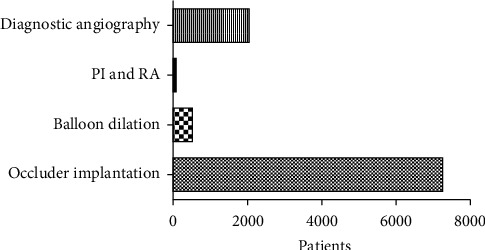
Operation diagram. PI = pacemaker implantation; RA = radiofrequency ablation.

As depicted in the flowchart (Figure 1), the database search identified 48 potentially qualified articles. Of these, 25 (9,738 patients in total, 4,602 males and 4,399 females) articles were determined to be eligible and included. Descriptive analyses of these articles are presented in Table 1. The patients were between 23 days and 14-year-old and weighed from 3 to 39 kg, including 2,004 cases of diagnostic angiography examination and 7,734 cases of interventional therapy. The interventional therapy cases were further subdivided into occluder implantation (7,215 cases), balloon dilation (468 cases), and pacemaker implantation and radiofrequency ablation (51 cases) (Figure 2).

### 3.2. Monitoring

In pediatric CHD interventional cardiac catheterization, all the included reports conventionally monitored the electrocardiogram (ECG), heart rate (HR), pulse oxygen saturation (SpO_2_), noninvasive blood pressure (NIBP), and breathing. Additionally, for the critical patients, blood gas and invasive blood pressure were monitored [8, 15, 17, 26–28] as well as partial tension in end-tidal carbon dioxide (PetCO_2_) [10, 11, 28] when necessary.

### 3.3. Anesthetic Methods

**Figure 3:**
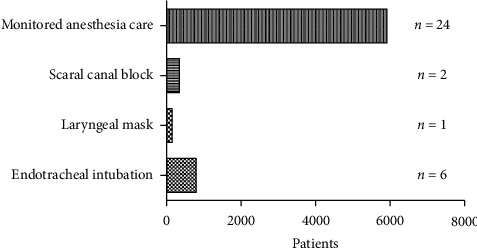
Anesthesia methods. *n* = number of articles.

In our study, Chinese anesthesiologists used endotracheal intubation [6, 8, 11, 19, 24, 28] or laryngeal mask [19] general anesthesia, monitored anesthesia care (MAC), and occasionally chose sacral canal block [11, 25]. Because Song et al. [11] did not indicate the number of cases of each anesthesia method in the article, the number of cases of endotracheal intubation, laryngeal mask, sacral canal block and MAC was 770, 120, 313, and 5,890, respectively (Figure 3). MAC was the most common option. In addition to those patients who were preoperatively considered for endotracheal intubation to control breathing, 31 (0.32%) patients underwent unexpected emergency endotracheal intubation in our research (Table 2).

### 3.4. Anesthetic Drugs

In our study, the reported anesthesia-related premedication, induction, and maintenance drugs are shown in Table 3, a variety of anesthesia-related drugs were used either alone or in combination, and 12 articles reported the use of preoperative drugs such as atropine, anisodamine, penehyclidine, diazepam, pethidine, morphine, and midazolam. Induction drugs like ketamine, midazolam, fentanyl, propofol, scopolamine, and penehyclidine were reported in intravenous anesthesia in 23, 19, 11, 4, 4, and 3 articles, respectively.

### 3.5. Complications

In our study, there were a total of 722 (7.41%) cases of anesthesia-related complications (Table 4), and the incidence of dysphoria was 2.09% (204 patients reported in 6 articles). Respiratory- and airway-related adverse events were the most common anesthesia-related complications and occurred in 3.88% (a total of 378 patients in 20 articles), including respiratory depression, cough, bronchospasm, laryngospasm, increased respiratory secretion, and airway obstruction. Respiratory depression included ① a low respiratory rate (RR) (<12 breaths/min), ② apnea lasting more than 15 seconds, ③ a >20% drop in SpO_2_, ④ an oxygen partial pressure (PO_2_) ≦40 mmHg, and ⑤ a PetCO_2_ >50 mmHg. The incidence of procedure-related complications was 12.14% (1,182 cases), the highest incidence was arrhythmia, and the second highest was hypotension (Table 5). Details of the fatal cases are shown in Table 6.

## 4. Discussion

Cardiac catheterization remains the gold standard for diagnosis and management in multiple forms of CHD [2]. During interventional cardiac catheterization, the patients should be quiet, cooperate, immobile, and with stable hemodynamics, and unobstructed airways and hypoxia should be avoided; consequently, anesthesia is often required. Ramamoorthy et al. [30] reported that anesthetic risk is higher in pediatric cardiac patients than in the general pediatric population.

Messeha and El-Morsy [31] reported that anxiety and psychological trauma due to maternal deprivation were major challenges in pediatric anesthesia. Preanesthetic medication decreases this anxiety and psychological trauma and facilitates the induction of anesthesia without delaying recovery. In this study, 12 articles reported the use of preoperative drugs (Table 3), focused on sedation, and reduced respiratory secretions. In the meta-analysis of Peng et al. [32], they compared dexmedetomidine premedication with midazolam or ketamine premedication or placebo in children, the methods of administering dexmedetomidine premedication including IM, PO, IV, and intranasal instillation, and suggested that dexmedetomidine is superior to midazolam premedication because it resulted in enhanced preoperative sedation and decreased postoperative pain.

In our study, when patients entered the cardiac catheterization laboratory, ECG, HR, SpO_2_, and NIBP were routinely monitored, and close attention was paid to their breathing. PetCO_2_ and blood gas were further monitored when necessary. PetCO_2_ can detect the presence or absence of air exchange as well as airway obstruction, hypoventilation, and apnea, and it has been a standard operating room method of monitor for years. Accordingly, there is increasing interest in the use of PetCO_2_ outside the operating room [33, 34], and newer sedation guidelines encourage its use [35]. Routine use of capnography has reduced anesthesia-related adverse outcomes [36]. When using capnography, cyanotic heart diseases patients warrant special attention. Friesen and Alswang [37] reported that PetCO_2_ correlated well with PaCO_2_, but besides cyanotic heart disease patients, these patients needed blood gas analysis.

Singh et al. [38] reported that nontracheal intubation general anesthesia is commonly used in the interventional cardiac catheterization of pediatric patients with CHD. In our study, Chinese anesthesiologists mostly chose monitored anesthesia care (MAC), and the greatest benefits of spontaneous breathing in MAC are short recovery time and promotion of venous return. Fewer anesthesiologists chose general anesthesia with controlled breathing, while mechanical ventilation allows for the control of PaCO_2_, regulates pulmonary vascular resistance (PVR), and does not involve airway obstruction; the depth of anesthesia is easily regulated and deep enough to blunt the reflexes to painful stimuli and ensure immobility [39]. However, positive pressure ventilation reduces venous return, alters the flow across valves and shunts, decreases the metabolic rate, decreases oxygen consumption, and further alters hemodynamics [40]. Therefore, most anesthesiologists choose MAC for the children with CHD during cardiac catheterization. The anesthesia induction method should be selected according to age, premedicant dosage, venous channel existence, lesions type, blood vessel function, and the possible response to different anesthetic drugs. And Behnaz et al. [41] reported the effect of sevoflurane and propofol on pulmonary arterial pressure during cardiac catheterization in children with CHD. In our study, intravenous induction was most commonly used. Ketamine often used in combination with propofol, midazolam, atropine, or penehyclidine usually provides a stable hemodynamic and respiratory profile with good recovery time and minimal delirium [42, 43]. In brief, we found that many different drugs combinations have been used safely and successfully with general anesthesia and sedated patients. The anesthesiologist must understand the limitations of each method and has a firm grasp of the basic knowledge of circulation physiology characteristics of pediatric patients with CHD. When physicians prepare to puncture the femoral artery or vein, anesthesiologists should increase the depth of anesthesia and the physician should administer 1% lidocaine local anesthesia at the puncture site.

In this study, the incidence of anesthesia-related complications was 7.41% (722 patients in Table 4), slightly higher than the 6% reported by Tokel et al. [44] and Behnaz et al. [45] perhaps because Tokel et al. only collected 2,662 patients reported from a single center over a five-year period. The incidence of procedure-related complications was 12.14% (1,182 cases reported in 15 articles); of these, arrhythmia and hypotension occurred in 9.24% (900 cases reported in 9 articles) and 2.21% (215 cases reported in 4 articles), respectively. Fuwai Hospital reported [46] that, from 1986 to 2009, a total of 6,029 patients with CHD were treated by interventional therapy, the incidence of procedure-related complications was 388 cases (6.44%) (in addition to arrhythmias), and the mortality rate was 0.08% (5 patients). Tokel et al. [44] reported that the incidence of adverse events in cardiac catheterization was 20%, with a mortality rate of 1.4%.

In cardiac catheterization, the arrhythmias were usually transient and included premature ventricular beats (polygenesis or synchrony), short episodes of ventricular tachycardia, atrial arrhythmias, ventricular tachycardia, atrioventricular block, and bradycardia. The majority of arrhythmias were related to stimulation of the atrial or ventricular wall by the catheter, and when the catheter was withdrawn immediately, the arrhythmia disappeared in most cases. If the arrhythmia remained unresolved, some relevant antiarrhythmic drugs should be considered, such as lidocaine, atropine, propafenone, and supplementary volume. Almost all sedative drugs cause peripheral vascular dilatation, plus the patient loses blood during femoral arteriovenous puncture, which commonly leads to intraoperative hypotension. The appropriate treatment method is to replenish volume. Other procedure-related complications included occluder detachment, emergency open heart surgery, wire looping or kinking, pericardial tamponade, heart failure, and pulmonary edema. These complications are relatively serious; thus, they need to be recognized quickly and brought to the physician's attention.

In our study, 25 (0.26%) patients experienced cardiac arrest, and recovery occurred after timely and effective cardiopulmonary resuscitation. Boston Children's Hospital [47] reported that the risk of cardiac arrest was 0.96% and was associated with procedural and system factors.

Fortunately, death is uncommon. Five patients died in our group, a mortality rate of 0.05% (Table 6), and all the cases involved were complex cyanotic patients with CHD undergoing right heart operations. Bergensen et al. [48] reported a mortality rate of 0.29% among 3,855 cases from six institutions, seven out of the eleven deceased patients were neonates, and cyanotic patients had an increased risk of mortality. Death after cardiac catheterization seems to be more related to the patient's general status than with the procedure itself.

## 5. Conclusions

For pediatric patients with congenital heart diseases who had undergone cardiac catheterization procedure in China, arrhythmia and hypotension are the most common procedure-related complications. Monitored anesthesia care is the commonly used anesthesia methods, and dysphoria, cough, nausea, vomiting, and respiratory depression are frequent complications associated with anesthesia.

## Figures and Tables

**Table 1:** Characteristics of the included articles.

Articles	Research institutions	Province	*N*	Operation
Wang, 2001 [5]	Fuwai Hospital	Beijing	369	E
Meng, 2016 [6]	Shenzhen Children's Hospital	Guangdong	324	T
Chang, 2007 [7]	Armed Police Corps Hospital of Shanxi	Shanxi	519	T
Lin, 2010 [8]	Anzhen Hospital	Beijing	1192	T
Wang, 2012 [9]	The First People's Hospital of Huaihua	Hunan	104	T
Huang, 2014 [10]	Guangxi Zhuang Autonomous Region People's Hospital	Guangxi	120	T
Song, 2008 [11]	Shijiazhuang First People's Hospital	Hebei	2645	E and T
Zhang, 2015 [12]	Kunming Yan'an Hospital	Yunnan	1000	T
Zhang, 2010 [13]	Shandong Qufu People's Hospital	Shandong	384	T
Gao, 2010 [14]	First Hospital of Hebei Medical University	Hebei	168	T
Zhang, 2008 [15]	Hunan Children's Hospital	Hunan	136	T
Chen, 2010 [16]	Maoming People's Hospital	Guangdong	108	T
Ma, 2012 [17]	People's Liberation Army Hospital 474	Xinjiang	124	T
Yin, 2010^*∗*^ [18]	Zhengzhou Institute of Cardiovascular Diseases	Henan	121	E and T
Xue, 2012 [19]	Beijing Airforce General Hospital	Beijing	236	T
Zhang, 2006 [20]	First Affiliated Hospital of Guangxi Medical University	Guangxi	100	T
Lin, 2010 [21]	Sixth Affiliated Hospital of Guangxi Medical University	Guangxi	409	T
Zhang, 2018 [22]	Guizhou Provincial People's Hospital	Guizhou	109	E and T
Huang, 2016 [23]	Gaozhou People's Hospital	Guangdong	100	T
Cheng, 2009 [24]	Bethune International Peace Hospital	Hebei	200	T
Zhou, 2006^*∗*^ [25]	Third Xiangya Hospital of Cental South University	Hunan	616	T
Li, 2011 [26]	Guizhou Provincial People's Hospital	Guizhou	165	T
Yao, 2010 [27]	Yulin First People's Hospital	Guangxi	125	T
He, 2010 [28]	First Affiliated Hospital of Tsinghua University	Beijing	139	E
Wang, 2003 [29]	Shanghai Children's Medical Center	Shanghai	153	E

^*∗*^Child sex was not reported; *E* = examination; *T* = treatment; *N* = sample size.

**Table 2:** Reasons for unexpected endotracheal intubation.

Possible reasons	Cases
Bawl violently, swallow large amounts of air, bloat, cough violently, vomiting	3 [5]
Pulmonary hypertensive crisis, induced by contrast	1 [5]
Vomiting, dysphoric, respiratory depression	1 [10]
Air embolism	1 [13]
Facial cyanosis, limb stiffness	2 [14]
Increased respiratory secretion, cough, bronchospasm, laryngospasm	8 [17, 18, 26–28]
Atrioventricular block, heart rate 50 beats per min	1 [17]
Respiratory depression, convulsion	6 [24]
Apnea, anoxic spells	7 [28]
Cardiac arrest	1 [29]

**Table 3:** Drugs used in anesthesia for cardiac catheterization.

Drugs	Premedication	Induction	Maintenance
Atropine	10–20 *μ*g/kg, IM [8, 10, 16, 24, 25]	0.01 mg/kg, IV [18, 19, 27]	—
Anisodamine	0.01 mg/kg, IM [9]	—	—
Atracurium	—	0.3 mg/kg, IV [24]	—
Cisatracurium	—	—	0.05–0.1 *μ*g/kg/min, CI [10]
Dezocine	—	0.1 mg/kg, IV [23]	—
Diazepam	0.1 mg/kg, IM/IV [9, 21]	—	—
Dolantin	1–1.5 mg/kg, IM [8]	—	—
Etomidate	—	0.1–0.2 mg/kg, IV [10, 14]	8–10 *μ*g/kg/min, CI [6, 10]
Fentanyl	—	1–3 *μ*g/kg, IV [9–11, 15, 17–19, 22, 24, 26, 27]	—
Granisetron	—	0.4 *μ*g/kg, IV [7]	—
Haloperidol	—	—	0.15–0.3 mg/kg, IM [5]
Isoflurane	—	—	1–3%, inhalation [9, 15]
Ketamine	—	3–8 mg/kg, IM [8, 19, 20, 24]	6–8 mg/kg, IM [5]
Ketamine	—	1-2 mg/kg, IV [5, 7, 10–18, 21–23, 25–28]	1-2 mg/kg, IIV [8, 9, 22, 26, 28]
Ketamine	—	—	0.9-6 mg/kg/h, CI [14, 21, 23, 24]
Morphine	0.1–0.2 mg/kg, IM [15]	—	—
Midazolam	0.1 mg/kg, IV [24]	50–150 *μ*g/kg, IV [7, 9, 12, 13, 15, 17–19, 22, 24, 26, 28]	—
Midazolam	—	0.1 mg/kg, IM [27]	—
Promethazine	1 mg/kg, IM [8, 28]	—	—
Penehyclidine	0.1–0.15 mg/kg, IM [15]	0.02–0.04 mg/kg, IV [17, 18, 27]	—
Pethidine	0.5-1 mg/kg, IM [20, 28]	—	—
Phenobarbital	2 mg/kg, IM [25]	—	—
Propofol	—	1-2 mg/kg, IV [12, 13, 21, 26]	1–9 mg/kg/h, CI [6, 7, 9, 11, 15, 17, 18, 20, 21, 23–25]
Propofol	—	—	2.5–4 *μ*g/ml, TCI [19]
Rocuronium	—	0.6 mg/kg, IV [10]	—
Remifentanil	—	—	4 ng/ml, TCI [19]
Remifentanil	—	—	0.05–0.15 *μ*g/kg/min, CI [10, 20]
Scopolamine	5–10 *μ*g/kg, IM [8, 13, 19–21, 28]	5–20 *μ*g/kg, IV [5, 7, 14, 17]	—
Sevoflurane	—	—	1%, inhalation [16]
Vecuronium	—	0.1 mg/kg, IV [15]	—

IM = intramuscular injection; IV = intravenous injection; CI = continuous infusion; IIV = intermittent IV; TCI = target-controlled infusion.

**Table 4:** Anesthesia-related complications.

Complications	Cases	Incidence (%)
Choking cough	128 [5, 19, 24]	1.31
Nausea, vomiting	129 [6, 9–12, 14, 16, 20, 23]	1.32
Respiratory depression	128 [5, 7, 9, 11–14, 16, 20, 23, 28, 29]	1.31
Bronchospasm	1 [11]	0.01
Tracheal tube detachment	1 [11]	0.01
Laryngospasm	17 [11, 17, 18, 26, 27]	0.17
Airway obstruction	72 [11, 12, 15, 22, 24]	0.74
Phrenospasm	11 [13]	0.11
Increased respiratory secretion	20 [11]	0.21
Dysphoria	204 [9, 12, 16, 19–21]	2.09
Local anesthetic poisoning	1 [11]	0.01
Illusion, nightmare	6 [9–11, 16]	0.06
Diplopia	4 [16]	0.04

**Table 5:** Procedure-related complications.

Complications	Cases	Incidence (%)
Arrhythmia	900 [5, 7, 13, 15, 17, 19, 23, 28, 29]	9.24
Cardiac arrest, ventricular fibrillation	25 [5, 13, 28, 29]	0.26
Hypotension	215 [7, 11, 14, 29]	2.21
Pulmonary hypertension	11 [11]	0.11
Emergency surgery	10 [5, 15, 18, 26]	0.10
Allergy	5 [9, 11, 16, 28]	0.05
Heart failure, pulmonary edema	5 [11]	0.05
Pericardial tamponade	2 [11]	0.02
Wire winding, wire kink	3 [11, 29]	0.03
Death	5 [5, 11, 18]	0.05
Air embolism	1 [13]	0.01

**Table 6:** Fatal cases.

Age (month)	Operation	Diagnosis	Intraoperative	Death reason
/[5]	RTC	TECD, PH	PHC	RVOTS, CA
8 [11]	RTC	TOF	CS, AHA	VF, CI
5 [11]	RTC	DORV, VSD, AS, PH	CS, PHC	VF, CI
15 [11]	PVBD	PS	AHA	RVOTS
/[18]	RTC	TECD, PH	PHC	—

RTC = right heart catheterization; PVBD = pulmonary valve balloon dilation; TECD = total endocardial cushion defect; PH = pulmonary hypertension; TOF = tetralogy of Fallot; DORV = double outlet right ventricle; VSD = ventricular septal defect; AS = aortic stenosis; PS = pulmonary stenosis; PHC = pulmonary hypertension crisis; CS = cardiogenic shock; CA = cardiac arrest; RVOTS = right ventricular outflow tract spasm; AHA = acute hypoxia attack; VF = ventricular fibrillation; CI = cardiac insufficiency.

## Data Availability

The data used to support the findings of this study are present within the article.
